# Synthesis of Fatty Acid Esters of Selected Higher Polyols Over Homogeneous Metallic Catalysts

**DOI:** 10.1007/s11746-016-2840-7

**Published:** 2016-05-09

**Authors:** Janusz Nowicki, Dorota Stańczyk, Jolanta Drabik, Jan Mosio-Mosiewski, Piotr Woszczyński, Marek Warzała

**Affiliations:** Institute of Heavy Organic Synthesis “Blachownia”, Energetyków 9, 47-225 Kędzierzyn-Koźle, Poland; Institute for Sustainable Technologies, National Research Institute, K. Pulaskiego 6/10, 26-600 Radom, Poland

**Keywords:** Esterification, Fatty acids, Pentaerythritol, Dimer TMP, Organometallic catalysts

## Abstract

Studies on the synthesis of esters of natural origin fatty acids (oleic acid) and a branched synthetic isostearic acid derived from oleic acid with commercially available selected higher polyols in the presence of homogeneous metallic catalysts have been carried out. The effects of the synthesis temperature, molar ratio and the catalysts amount have also been studied. It was shown that higher fatty acid conversion and selectivity to tri- and tetraesters were obtained for organotin catalyst Fascat 2003, which was used as the esterification catalyst. Anti-wear test confirmed good tribological properties of the obtained esters.

## Introduction

Esters of fatty acids of vegetable origin with higher polyols such as pentaerythritol (PT) or trimethylolpropane dimer (DiTMP) containing four OH groups are one of the most important biocomponents of hydraulic oils and greases. Among them the most popular and widely described in the literature is pentaerythritol tetraoleate.

When considering the potential directions of the use of those esters in addition to viscosity parameters also essential is their pour point. These both parameters are highly dependent on the kind of fatty acid used. Table 1 shows the effect of the type of fatty acid on the pour point of various pentaerythritol esters. From Table 1 it is evident that a lower pour point can be obtained for mixed long chain fatty acids (C_18_) esters of pentaerythritol. Commercial oleic acid ester of pentaerythritol (PETO-A Grade) has a slightly worse pour point (approx. −30 °C).

**Table 1:** Pour points of pentaerythritol esters of various fatty acids [1]

	Pour point, °C
PT esters of branched C_9_ acids	+30
PT esters of linear C_9_ acids	+8
PT esters of branched C_8_ acids	+8
PT esters of branched C_18_ acids	−38
PT mixed esters of linear and branched C_18_ acids	−48

**Scheme 1:**
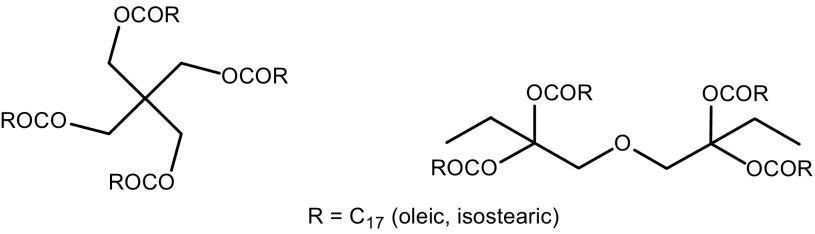
Fatty acid esters of pentaerythritol and TMP dimer synthesized in this study

Pentaerythritol tetraoleate is characterized by high lubricating properties, high viscosity index, high biodegradability and good flame retardancy. These features make pentaerythritol tetraoleate a valuable compound used in the manufacture of fire-resistant hydraulic fluids of ISO 68 grade, VG-68 grade engine oils, oils for gas turbines and oils used in metalworking [2–7]. Esters of pentaerythritol and C_5_–C_10_ fatty acids are also used as components of base oils for the aviation industry. Properties similar to pentaerythritol tetraoleate can be expected from fatty acid esters of TMP dimer. TMP dimer is a polyol that also contains four OH groups, and is readily available in industrial quantities (Scheme 1). Pentaerythritol esters of higher fatty acids have also been widely used as plasticizers and in cosmetic formulations as emollients [1, 7–10]. Of a particular importance, as a component of cosmetic gels, is pentaerythritol tetraisostearate.

However, the literature about the methods of the synthesis of fatty acid esters and higher polyols, in particular pentaerythritol esters, is very limited. A basic method of the synthesis of pentaerythritol fatty acid esters is an esterification reaction carried out in the presence of an acidic catalyst. The synthesis of pentaerythritol tetraoleate with the use of sulfonic homogeneous catalysts have been described in the patent literature [11, 12]. The authors described a method using *p*-toluenesulfonic acid or lanthanum salt of methanesulfonic acid as a catalysts. For oleic acid to pentaerythritol molar ratio 4.4:1, at a temperature of 160 °C, after 4 h 96 % polyol conversion is achieved. The crude ester is then purified by molecular distillation to give the final product with 80 % yield. The use of p-toluenesulfonic acid as an esterification catalyst was also described by Langeroodi *et al*. [13]. The synthesis was carried out at 200 °C for an equimolar ratio of the fatty acid to OH groups. The authors obtained, however, a dark-colored product, which required additional purification steps. A similar method was also described by Yao [14]. In the synthesis benzene as additional solvent has been used, what helped to remove the esterification water as a heteroazeotrope. The esterification reaction was carried out with an excess of oleic acid, which was removed by fractional crystallization. The synthesis of esters of the higher polyols, including pentaerythritol, using toluene as the solvent was described in detail by Nagendramma *et al*. [15, 16] and Zhang *et al*. [17]. There have also been methods of synthesis described with the use of various heterogeneous catalysts. Gao *et al*. reported a method of synthesis of pentaerythritol esters with the use of ZrO_2_–Al_2_O_3_/SO_4_^2−^ superacid as a heterogeneous catalyst [18]. The authors used a large excess of fatty acid (5.6:1). After 4 h of reaction the ester was obtained with a high yield, exceeding 99 %, but the removal of the excess of fatty acid required high vacuum distillation. Recently Ravasio *et al*. reported the use of SiO_2_–Al_2_O_3_, SiO_2_–ZrO_2_ and SiO_2_–TiO_2_ amorphous oxides as effective catalysts for the synthesis of polyol fatty acid esters [19]. For SiO_2_–ZrO_2_ the highest pentaerythritol conversion (99 %) and selectivity to tetraester were achieved. The authors described this method as “zero waste”, but the use of heterogeneous catalyst before next re-use requires washing with harmful acetone. Itsikson, in turn, studied the esterification reaction of pentaerythritol and oleic acid with the use of tributyl phosphate, tributyl phosphite, sodium sulfate and activated carbon impregnated with H_2_SO_4_ as a catalyst [20]. The reaction was carried out with 10 % molar excess of oleic acid, which was subsequently removed by distillation under high vacuum. The final ester also required purification by distillation under very high vacuum. The yield of the crude ester varied from 99.5 % (phosphates) to 84.4 % (activated carbon) and of the final product from 85 to 67 %, respectively. The use of tributyl phosphate as the catalyst for the synthesis of esters of pentaerythritol was also described by Guan *et al*. [21]. At a temperature of 220 °C, for the molar ratio of fatty acid to OH = 1.1:1 after 5 h they obtained the ester with a yield of 96 %. Akerman *et al*. described a “clean” method of synthesis.

Fatty acids esters (oleate, isostearate) of pentaerythritol are manufactured on an industrial scale, but the main method of their synthesis is based on homogeneous sulfonic acid catalysts. This method requires purification of crude ester by distillation under high vacuum. In this paper we have described an efficient method for the synthesis of esters of fatty acids (oleic and isostearic) and higher polyols (pentaerythritol and TMP dimer) with the use of homogeneous organometallic catalysts. The resulting esters do not require purification by high vacuum distillation, but only a standard washing and final drying, which makes the described method applicable for use on an industrial scale successfully.

## Experimental

### Materials

Oleic acid 90+ % was purchased from Croda Int. as Priolene 6936. This product is a mixture of distilled fatty acids and contains <3 wt% of <C_18_ acids, 2 wt% of <C_18:0_ acid, 92 wt% of C_18:1n9_ acid, 3 wt% of C_18:2n6_ acid, 0.3 wt% of C_18:3n3_ acid and <1 wt% of >C_18_ acids (GC). Isostearic acid was purchased from Unichema (UK) as Prisorine 3603. This product contains ca. 83 %wt of branched and saturated acids C_16_–C_22_. Pentaerythritol was purchased from Alfa Aesar. Trimethylolpropane dimer was purchased from Perstorp (Sweden).

As catalysts we used selected commercially available homogeneous metallic Sn, Ti and Zr catalysts. All of the catalysts used are listed in Table 2.

**Table 2:** List of organometallic catalysts used in study

Catalyst	Trade name	Abbreviation	Supplier
Di(*n*-butyl)tin oxide	–	Bu_2_SnO	Alfa Aesar
*n*-Butyltin hydroxide oxide	–	BuSn(O)OH	Alfa Aesar
Tin bis-(2-ethylhexanoate)	Fascat 2003	F 2003	PMC
Butyl tin tris-(2-ethylhexanoate)	Fascat 4102	F 4102	PMC
Tetra-(*n*-butyl) titanate	Tyzor TBT	T TBT	DuPont
Tetra-(isopropyl) titanate	Tyzor TPT	T TPT	DuPont
Tetra-(*n*-butyl) zirconate	Tyzor NBZ	T NBZ	DuPont

## Methods

### General Procedure for Esterification of Polyols

A 200-mL glass reactor, equipped with a mechanical stirrer, an electronic temperature control system, a nitrogen purge glass capillary and a receiver to collect esterification water was charged with 180 g (0.6 mol) of oleic acid and 0.15 mol of corresponding polyol (pentaerythritol, TMP dimer). The reactor was heated up to 150 °C and then a specific amount of catalyst was poured. After stabilizing the flow of nitrogen, the temperature was raised to 220 °C. The reaction mixture was stirred for 6 h. During synthesis the esterification water was collected in the receiver. The crude ester was washed with 5 wt% K_3_PO_4_ aqueous solution and with water to remove residual acidic impurities, and then it was dehydrated under slightly reduced pressure to remove residual water.

### Analysis of Esterification Products by Gas Chromatography

Products were analyzed by gas chromatography with a head-space system using an HP 5890 Series II chromatograph equipped with flame ionization detector, capillary column, Ultra-high-2 (5HT), l = 15 m, *d* = 0.32 mm. All samples were transformed into silane derivatives with BSA (*N*,*O*-bis(trimethylsilyl)acetamide). Instrument settings: injector temp. 360 °C, detector temp. 380 °C. Temperature programming: initial isotherm 100 °C—1 min, 100–380 °C—gradient 15 °C/min, final isotherm 380 °C—3 min. Carrier gas: argon—1.8 ml/min. Quantitative interpretation of the results was performed by internal standardization for all components assuming a correction factor equal to 1. The conversion rate of acid was calculated as the ratio between the amount of the acid consumed in the synthesis to a total amount of the acid used in the synthesis. Resultant ratios were then converted to percentages. In calculations, for simplification, as isostearic acid C_18:0_ acid was adopted.

### Determination of the Physicochemical Properties of Polyol Esters

Kinematic viscosity measurements were made at 40 and 100 °C using calibrated Ubbelohde Viscometer tubes. Viscosity and viscosity index (VI) were calculated using ASTM D 445 and ASTM D 2270 methods, respectively. All viscosity measurements were run in duplicate and the average value was reported. Pour points were determined according to standard ISO 3104. All runs were carried out in duplicate. Sample temperature was measured in 3 °C increments at the top of the sample until it stopped pouring. Acid value was determined according to standard ISO 660:1996. The saponification value was determined according to standard ISO 3657:2013–2010. The hydroxyl value was determined according to standard DIN 53240-1. Color of esters (Gardner) was determined on a Hach-Lange Lico 100 colorimeter.

### Determination of Tribological Properties of Polyol Esters

Lubricating properties of the base oil and the influence of the modifiers on these properties were assessed using the T-02U universal machine. The T-02U universal four-ball testing machine is intended for determination of extreme pressure (EP), antiwear (AW) and antifriction properties of lubricants and engineering materials, as well as determination of the tendency of lubricants and engineering materials to produce surface fatigue failures (pitting). All the tests may be carried out at an elevated temperature. The T-02U machine determines EP and AW properties in accordance with the standards: ISO 20623, ASTM D 2783, ASTM D 2596, ASTM D 4172, ASTM D 2266, IP 239, PN-76/C-04147 [22].

The wear standardized measurements, the average wear scar diameter (d), limiting load of the wear (Goz) were determined. The average wear scar diameter and limiting load of the wear, which characterizes the antiwear (AW) properties of the tested oils, were determined after the 3600-s runs at a constant rotational speed (1450 rpm) and constant load (392.4 N). Assessment of the antiseizure based on scuffing load (Pt) and limits seizure load (poz) were determined according to the standardized methods. The scuffing load, reflecting the extreme-pressure (EP) properties of the tested oils, was determined after the 18-s runs at a constant rotational speed (500 rpm) and a step-wise increasing load that were performed until seizure of the test balls was observed. Determining the value of scuffing load for steel–steel friction joint (100Cr6/100Cr6) lubricated with investigated oils. Taken as a measure of resistance to scuffing load Pt at which a sharp increase in friction torque and limiting pressure of seizure poz characterized by the value of the loading force at which there has been a seizure of friction. The parameters values of the tribological test are given in Table 3. The balls used in this study were steel balls, 12.7 mm in diameter, with 60-65HRC hardness. The sample volume required for each test was 8 ± 2 ml. The wear produced on the three stationary balls is measured under a calibrated microscope and reported as the WSD (wear scar diameter) or calculated wear.

**Table 3:** The parameters of the tribological test

Parameter	*G*_oz_ (Nmm^−2^)	*P*_t_ (N)	*P*_oz_ (Nmm^−2^)
Rotational velocity of the upper ball	1450 rpm	500 rpm	500 rpm
Time	1 h	18 s	18 s
Test temp (°C)	20 ± 5	20 ± 5	20 ± 5
Initial load of the test couple	392.4 N	0 N	0 N
Final load of the test couple	–	7200 N	7200 N
Speed of load increase	–	409 N/s	409 N/s
Measurement	Wear scar diameter, limiting load wear, *G*_oz_	Scuffing load *P*_t_	Scar diameter, limiting seizure pressure *p*_oz_

## Results and Discussion

### Esterification of Fatty Acids

It is well known that progress of esterification reaction is significantly influenced by the efficiency of removal of the esterification water. In the case of low molecular weight alcohols (methanol, ethanol), which do not form heteroazeotropic mixtures with water, the process is difficult. In such cases the result is achieved by using a large excess of alcohol.

A more convenient case is for aliphatic alcohols >C_4_. They form heteroazeotropic mixtures with water, which facilitates removal of the esterification water. In the case of higher aliphatic alcohols >C_10_ and low volatility polyols such a process is not viable. Water is then removed by either using other solvents (e.g. toluene) or by running the esterification reaction at higher temperatures. The esterification of pentaerythritol, TMP dimer or similar polyols with of fatty acids requires using high temperatures.

This is because of the need for an effective removal of the esterification water. In addition, relatively high melting points of these polyols and their practical insolubility in the fatty acids causes the initiation of the esterification reaction to require temperatures above 150 °C. These requirements are perfectly met by homogeneous metallic catalysts, which are widely used in polyesters synthesis usually carried out at temperatures above 200 °C. Their activation occurs at temperatures of approx. 180 °C.

There are many of these types of catalysts available on the market, mainly organic Sn, Ti and Zr compounds like well-known C_3_–C_4_ titanates or zirconates as well as a more specific ones, such as tin bis(2-ethylhexanoate) or tin alkyl oxides.

**Fig. 1:**
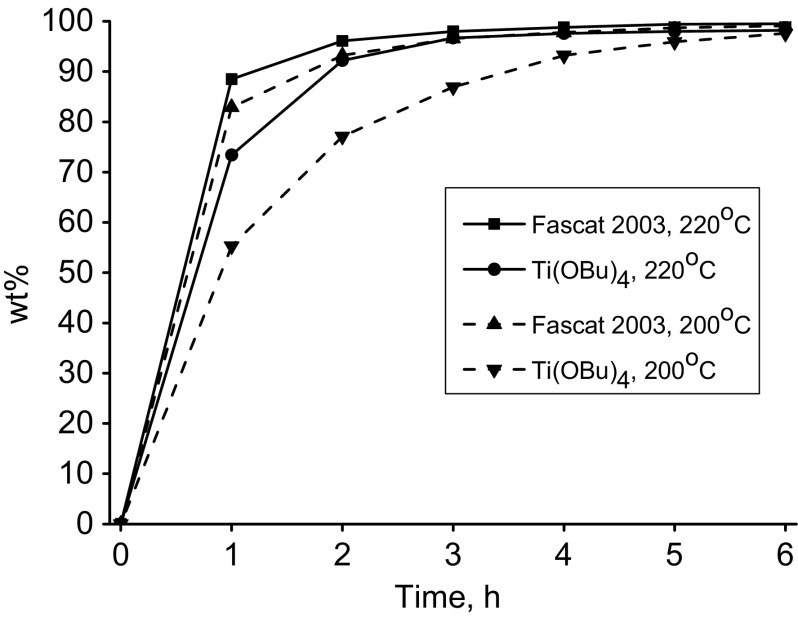
Oleic acid conversion into pentaerythritol oleate in the presence of Fascat 2003 and Ti(OBu)_4_ as catalyst. Reaction conditions: FA:OH mol. ratio (1:1), catalyst (0.63 wt%)

For evaluation of the activity of selected homogeneous metallic catalysts in the esterification reaction of pentaerythritol, a series of experiments with defined reaction parameters was carried out. In the first step as fatty acid we used oleic acid. On the basis of the results of the reaction products composition analysis, the effectiveness of selected catalysts was assessed. The results are given in Table 4. The results reported in Table 4 show that in the studied reactions the efficiency of used catalysts was varying. The most effective was the organotin catalyst Fascat 2003. The conversion of oleic acid was 98.5 %. A tetraester content in the crude product exceeded 85 wt% and a triester 9 wt%. The total content of pentaerythritol tri- and tetraesters was close to 97 wt%. Slightly worse results were obtained for tetrabutyl titanate (T TBT). Also, high conversion of oleic acid of 97 % has been achieved where the total content of tri- and tetraesters reached over 97 wt%. However, the crude product is characterized by a less favorable composition. The tetraesters content did not exceed 85 wt% and the triester content was close to 13 wt%. Figure 1 shows the reaction of esterification of oleic acid with pentaerythritol at two temperatures −200 and 220 °C simultaneously comparing the effects of the two best catalysts for this reaction. Figure 1 clearly shows the difference in the activity of the two tested catalysts and confirmed a higher activity of the organotin catalyst Fascat 2003. Fascat 2003 is a neutral and non-corrosive liquid catalyst widely used in polyesters manufacturing to increase the rate of resin formation and to increase the crosslinking speed in various coating applications. Fascat 2003 catalyst can be also used for the formation of esters from acids and alcohols. Because of a very small amount of catalyst used in synthesis the final ester products will have low tin content. Fascat 2003 catalyst does not require extensive or rigorous handling conditions and can be charged at any point during the reaction. As reported by Blank, Fascat 2003 is safer than other organotin catalysts. The toxicity concerns about organotin compounds does not apply to stannous carboxylates [23].

**Table 4:** The results of the esterification of oleic acid with pentaerythritol (GC, wt%)

	Catalyst						
	Bu_2_SnO	BuSn(O)OH	F 2003	F 4102	T TPT	T TBT	T NBZ
Pentaerythritol	tr.	tr.	tr.	tr.	tr.	tr.	tr.
Oleic acid	3.9	6.4	1.4	3.0	2.8	1.9	2.2
PT monoester	1.2	4.4	–	1.1	1.5	0.2	–
PT diester	4.1	8.7	1.5	5.8	6.8	2.0	1.6
PT triester	23.3	7.5	4.3	25.8	14.3	12.4	14.7
PT tetraester	56.7	62.5	91.2	62.3	72.9	83.0	79.3
Other	10.8	10.4	2.9	2.0	1.7	0.5	2.2
FA conv., %	96.1	93.6	95.5	97.0	97.2	98.1	97.8
Ʃtri- and tetraesters	80.0	70.0	95.5	88.1	87.2	95.4	94.0

Reaction conditions: oleic acid—100 g, pentaerythritol—12 g, catalyst—0.7 g, temp.—220 °C, time—6 h

**Fig. 2:**
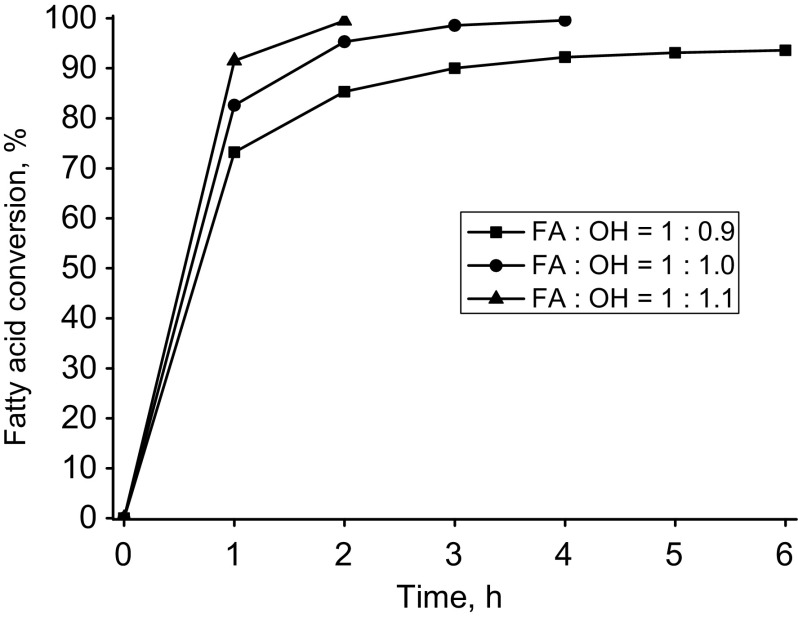
Oleic acid conversion into pentaerythritol oleate depending on the FA:OH molar ratio. Reaction conditions: (temp. = 220 °C, Fascat 2003, 0.63 wt%)

**Fig. 3:**
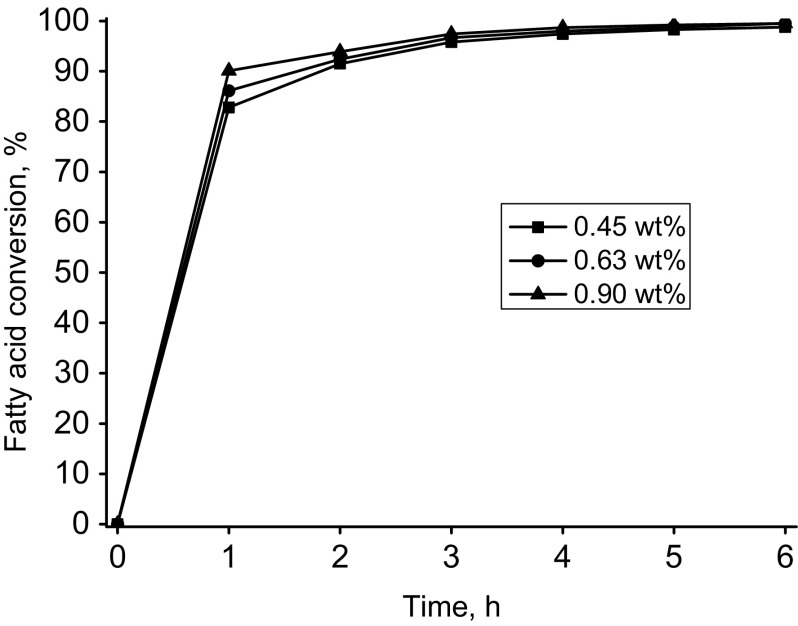
Oleic acid conversion into pentaerythritol oleate depending on the catalyst amount. Reaction conditions: (temp. 220 °C, Fascat 2003, FA:OH mol. ratio (1:1)

For the selected catalyst, the impact of the main parameters of the synthesis, i.e. molar ratio, temperature and amount of added catalyst were examined. Table 4 summarizes the results of esterification of oleic acid with pentaerythritol for different values of the molar ratio of the reactants. The effect of the molar ratio on fatty acid conversion has been also presented in Fig. 2. As expected, the molar ratio of oleic acid to pentaerythritol is important for both the product composition and the course of esterification reaction. Given the composition of esterification product the best result was obtained for equimolar ratio (FA:OH). In this reaction conditions conversion of oleic acid above 98 % was achieved. Crude (non-purified) product contained only 4.3 wt% of triester and above 91 wt% of tetraester (Table 4). Higher conversion of oleic acid (close to 99.5 %) was obtained for the molar ratio FA:OH of 1:1.1. High conversion is achieved after 2 h of the reaction, however a product with a definitely unfavorable composition is then made. Figure 3 shows the course of the esterification reaction showing the effect of the amount of added catalyst.

**Fig. 4:**
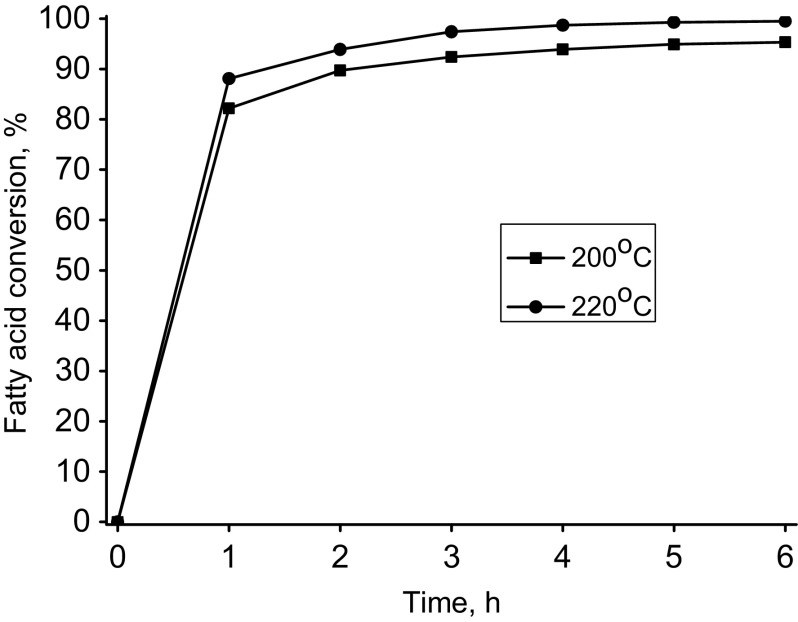
Oleic acid conversion into pentaerythritol oleate depending on reaction temperature. Reaction conditions: Catalyst (Fascat 2003, 0.63 wt%), FA:OH mol. ratio (1:1)

As can be seen, the amount of added catalyst has a relatively small effect on the course of the esterification. Figure 4 shows the influence of the reaction temperature on the reaction of esterification of oleic acid with pentaerythritol. The results shown in Fig. 4 indicate that increasing the temperature from 200 to 220 °C has a favorable, although not a very strong, influence on the course of the reaction. At higher temperature, however, one can get higher conversion of oleic acid and can even shorten the time of the synthesis. Figure 4 shows the results of esterification carried out with a smaller amount of the added catalyst. Increasing the amount of catalyst to 0.9 wt% has a small impact on the conversion (see Fig. 3).

**Fig. 5:**
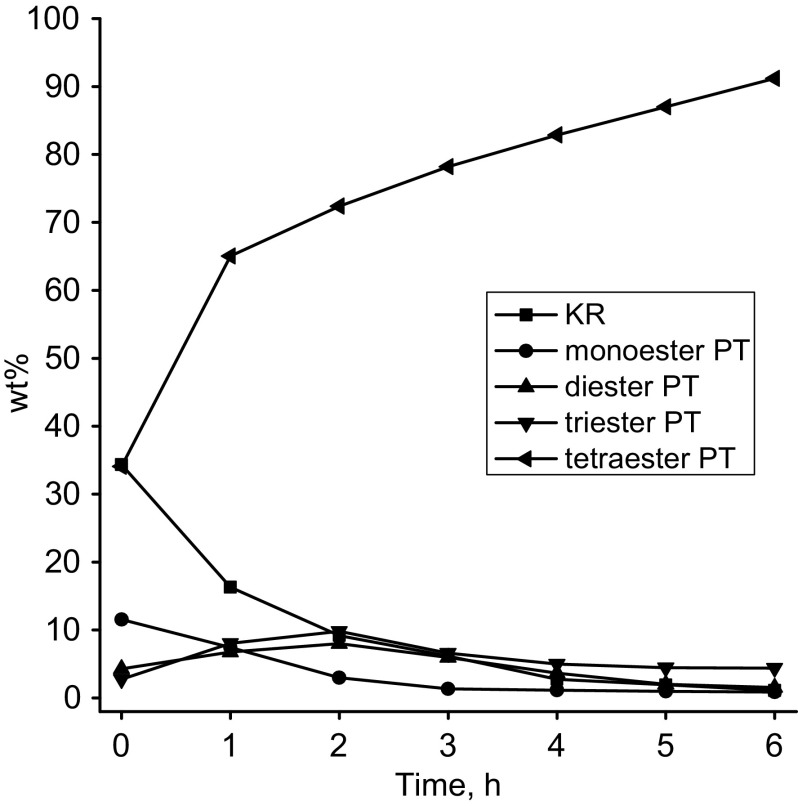
Compositions of esterification product of pentaerythritol with oleic acid. Reaction conditions: Temperature (220 °C) Catalyst (Fascat 2003, 0.63 wt%), FA:OH molar ratio (1:1)

**Fig. 6:**
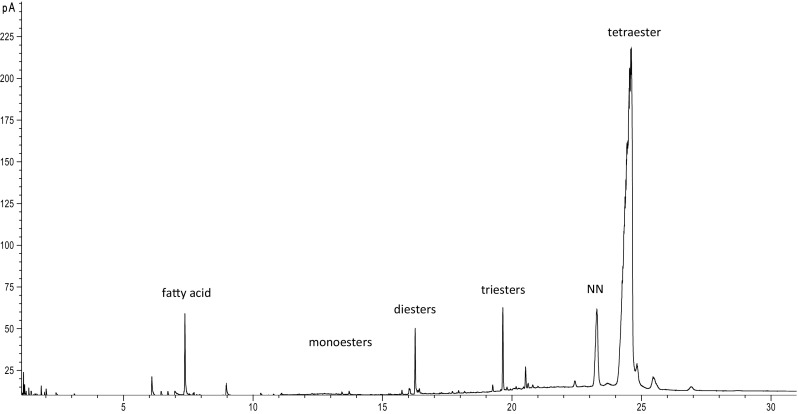
Representative GC chromatogram of pentaerythritol oleate synthesized in study. NN—unidentified compound

It can be concluded that it is preferable to use a smaller amount of the catalyst (0.63 wt% is preferred) and the esterification reaction may be carried out at a temperature of 220 °C. The Fig. 5 shows the course of the esterification reaction in terms of changes of the composition of the reaction mixture. Figure 5 clearly shows that the expected, preferred composition of the esterification product within the determined parameters of the synthesis is achieved in approx. 6 h. At an early stage of the synthesis, a rapid decrease in the content of oleic acid and a rapid increase in the content of tetraester are observed. Changes in the content of other esters are not so big. After 3 h of the reaction time the content of triester reaches a maximum value of nearly 20 % wt%, which, however, during further course of the reaction decreases to approx. 5 wt%. Figure 6 shows a representative GC chromatogram of pentaerithritol oleate.

Under the reaction conditions determined for the synthesis of pentaerythritol tetraoleate the synthesis of analogous ester of oleic acid and trimethylolpropane dimer was performed. TMP dimer is a polyol with four OH groups and can be regarded as an analog of pentaerythritol. As it has been previously mentioned the crude esterification product requires only purification to remove the residual fatty acid. For this purpose, the crude TMP dimer oleate was washed with a 5 wt% aqueous solution of K_3_PO_4_. The amount of K_3_PO_4_ used for washing was calculated as a three-fold molar excess relative to the acid number of the crude product. The purification washing was done at 50 °C for 30 min. After phase separation, the ester was washed once more with water to neutral pH, which was followed by reduced pressure distillation drying. Table 5 summarizes the compositions of the purified oleic and acid esters of pentaerythritol, and TMP dimer. The composition of TMP dimer tetraoleate was similar to the corresponding ester of pentaerythritol. The content of the sum of tri- and tetraester was 97.4 wt%, which is similar to pentaerythritol ester.

**Table 5:** Compositions of purified oleic acid and isostearic acid esters of pentaerythritol and TMP dimer (GC, wt%)

Fatty acid	Oleic acid		Isostearic acid	
Polyol	PT	DiTMP	PT	DiTMP
Polyol	–	–	–	–
FA	0.3	0.3	0.3	0.3
Monoesters	0.1	0.6	0.2	0.1
Diesters	0.3	2.4	2.5	2.1
Triesters	2.5	6.6	8.1	6.6
Tetraesters	94.2	89.0	88.9	89.3
Other	2.9	1.2	–	1.6
Σtri- and tetraesters	96.7	95.6	97.0	95.9
FA conversion	99.7	99.7	99.7	99.7

For the obtained esters of oleic acid, the basic physicochemical properties were determined including viscosity characteristics, viscosity index, acid number, and pour point. The results were compared with the values of the corresponding parameters for a commercial oleic acid esters of pentaerythritol (Table 6). The results summarized in Table 6 clearly show that pentaerythritol oleate obtained with the use of homogeneous organotin catalyst is characterized by similar physicochemical data compared to commercial pentaerythritol tetraoleate. However, this ester is manufactured by using a more demanding technology requiring molecular distillation under very high vacuum [11, 12]. The method described in this paper is free of such a problem. The ester of the TMP dimer has similar parameters to the analogous pentaerythritol esters. Its higher viscosity is caused by a higher molecular weight of the used polyol.

**Table 6:** Physicochemical characteristics of oleate and isostearate esters of selected polyols

Product	PT tetraoleate comm. grade	PT oleate	DiTMP oleate	PT isostearate	DiTMP isostearate
Appearance	colorless, clear liquid	yellow, clear liquid	yellow, clear liquid	yellow, clear liquid	yellow, clear liquid
Color (Gardner)	5	5	5	5	5
Viscosity, m^2^/s,					
40 °C	60–70	71.8	84.3	130.5	164.8
100 °C	12.5–13.5	13.2	14.6	18.3	20.9
Viscosity index	>180	189	176	157	147
*L*_A_, mg KOH/g	max. 1	0.2	0.2	0.2	0.3
*L*_S_, mg KOH/g	175	175	172	175	173
*L*_OH_, mg KOH/g	<10	7	5	9	10
Pour point, °C	≤−30	−22	−32	−26	−22

*L*_A_ acid value; *L*_S_ saponification value; *L*_OH_ hydroxyl value

Table 5 presents also the chemical composition of purified isostearic acid esters of pentaerythritol and the TMP dimer. On the market, there are several brands of isostearic acid. In our studies we used isostearic acid obtained from oleic acid, so it can also be considered as a raw material derived from renewable sources. Both oleic and isostearic acid esters of pentaerythritol are used in the cosmetic industry as binders; skin-conditioning agents and viscosity increasing agents [24]. The chemical compositions of isostearic acid esters shown in Table 4 does not differ significantly from the corresponding esters of oleic acid. Table 6 presents the results of determinations of physicochemical properties of isostearic acid esters. Isostearic acid esters of pentaerythritol are characterized by much higher values of kinematic viscosity, but also by lower, when compared to the corresponding esters of oleic acid, values of the viscosity index. In Table 7 are summarized the results of the four-ball test for fatty acid esters of selected polyols (lubrication tests).

**Table 7:** Tribological properties of fatty acid esters of selected higher polyols

	PT oleate	DiTMP oleate	PT isostearate	DiTMP isostearate
Wear scar d, mm	0.66	0.63	0.72	0.69
Limit. wear load, *G*_oz_, N/mm^2^	468	513.7	393.4	428.6
Scuffing load, *P*_t_, N	1200	1300	1250	1250
Limit. pressure of seizure, *p*_oz_, N/mm^2^	259.9	377.5	298.2	340

The results of the performed four-ball test of the esters obtained show that esters of fatty acids (oleic and isostearic) and pentaerythritol and the TMP dimer synthetized according to the adopted method, which used easily accessible organotin catalyst are characterized by good lubrication properties. Measured scuffing load of esters were at the level of 1200–1300 N and wear scar diameter at the level of 0.66–0.72 mm can be considered as good compared to published data (1600 N and 0.79 mm respectively), in particular wear scar diameter [16]. As can be seen in Table 7 lubrication properties of TMP dimer esters are better compared to the corresponding pentaerythritol esters. Physicochemical properties of esters indicated in Tables 6 and 7 clearly show that the described method for the synthesis of polyol esters using the specific organotin catalysts provides a convenient way to obtain products which are potentially useful for tribological and cosmetic applications.

## Conclusions

The esterification of pentaerythritol and TMP dimer with oleic acid and vegetable derived isostearic acid was carried out using selected homogenous organic Sn, Ti and Zr catalysts. In the studied reactions the best results were achieved for tin di(2-ethylhexanoate). For this catalyst the optimal synthesis parameters were determined, in particular, the fatty acid-polyol molar ratio, the reaction temperature, the reaction time and the amount of added catalyst. Under the most favorable conditions the highest conversion of fatty acid, close to 99 % was achieved, and additionally the sum of tri- and tetraesters was above 90 wt%. The described method of the synthesis is fully suitable for a technical scale, does not require a high vacuum distillation processes and allows one to obtain high-quality products.

A systematic evaluation of basic physicochemical and lubricating properties of the synthesized esters was also carried out. Viscosities of pentaerythritol esters were from 72.8 to 130.5 mm^2^/s at 40 °C and from 13.3 to 18.3 mm^2^/s at 100 °C. For TMP dimer esters were from 84.3 to 164.8 mm^2^/s at 40 °C and from 14.6 to 20.9 mm^2^/s 100 °C, respectively. All esters were characterized by a low pour point from −22 to −32 °C and by a good viscosity index, which was significantly better for the oleic acid esters (172–175).
